# Prospective Analysis of the Influence of Sport and Educational Factors on the Prevalence and Initiation of Smoking in Older Adolescents from Croatia

**DOI:** 10.3390/ijerph14040446

**Published:** 2017-04-20

**Authors:** Natasa Zenic, Djivo Ban, Sanja Jurisic, Mladen Cubela, Jelena Rodek, Ljerka Ostojic, Mario Jelicic, Antonino Bianco, Damir Sekulic

**Affiliations:** 1 Faculty of Kinesiology, University of Split, 21000 Split, Croatia; natasazenic@yahoo.com (N.Z.); djivo.ban@du.t-com.hr (D.B.); jelena.rodek@kifst.hr (J.R.); ljerka.ostojic@kifst.hr (L.O.); mariojelicic@yahoo.com (M.J.); 2 University of Dubrovnik, 20000 Dubrovnik, Croatia; 3 Faculty of Medicine, University of Mostar, 88000 Mostar, Bosnia and Herzegovina; sanjajurisic10@gmail.com (S.J.); mladen.cubela@gmail.com (M.C.); 4 Academy of Medical Sciences, 71000 Sarajevo, Bosnia and Herzegovina; 5 Sport and Exercise Research Unit, SPPF Department, University of Palermo, 90144 Palermo, Italy; antonino.bianco@unipa.it; 6 University Department of Health Care Studies, University of Split, 21000 Split, Croatia

**Keywords:** cigarettes, academic achievement, sports, puberty, association

## Abstract

The prevalence of smoking among Croatian adolescents is alarmingly high, but no previous study has prospectively examined the sport- and academic-factors associated with smoking and smoking initiation. This study aimed to prospectively examine the associations between scholastic (educational) achievement and sport factors and smoking in 16- to 18-year-old adolescents. This two-year prospective cohort study included 644 adolescents who were 16 years of age at baseline (46% females). Baseline testing was implemented at the beginning of the 3rd year of high school (September 2014) when participants were 16 years old. Follow-up testing was completed at the end of the fourth year of high school, which occurred 20 months later. The evaluated predictor variables were educational-achievement- and sport-related-factors. The outcome variables were (i) smoking at baseline; (ii) smoking at follow-up; and (iii) smoking initiation over the course of the study. We assessed the associations between predictors and outcomes using logistic regression models adjusted for age, gender, socioeconomic status, and conflict with parents. The educational variables were consistently associated with smoking, with lower grade-point-average (Baseline: odd ratio (OR): 2.01, 95% confidence interval (CI): 1.61–2.55; Follow-up: 1.59, 1.31–1.94), more frequent absence from school (Baseline: OR: 1.40, 95% CI: 1.19–1.69; Follow-up: 1.30, 1.08–1.58), and lower behavioral grades (Baseline: OR: 1.80, 95% CI: 1.10–2.89; Follow-up: 1.57, 1.03–2.41) in children who smoke. Adolescents who reported quitting sports were at greater odds of being smokers (Baseline: 2.07, 1.31–3.32; Follow-up: 1.66, 1.09–2.56). Sport competitive achievement at baseline was protective against smoking initiation during following two-year period (0.45, 0.21–0.91). While the influence of the educational variables on smoking initiation has been found to be established earlier; sport achievement was identified as a significant protective factor against initiating smoking in older adolescents. Results should be used in development of an anti-smoking preventive campaign in older adolescents.

## 1. Introduction

The prevalence of smoking in Croatian adolescents is alarming. In short, given that 33% of adolescents report smoking, Croatia is, together with Bulgaria (33%), Italy (37%), Romania (30%), and Slovakia (31%) among the EU countries with the highest prevalence of smoking in adolescents [1]. During the last 5–10 years, public health authorities have placed a special emphasis on the development of efficient preventive campaigns against smoking, but the prevalence of adolescent smoking has not decreased significantly over the last five years [1,2]. It is likely that the cultural heritage of this territory (i.e., a significant portion of former Yugoslavia was part of the Ottoman Empire for more than four centuries) has played a role in smoking being looked upon as a socially accepted behavior, and availability of tobacco products has played important role in the relatively stable adolescent smoking figures. The role of these factors has been supported in studies examining other countries within this region [3,4,5]. Also, it has been approximated that more than one-fourth of the tobacco market in Croatia originates from the black market [6], which has significantly diminished the potential effect of higher prices of tobacco because of the taxing (increase of approximately 17% during the last five years) on smoking prevalence. It is well known that smoking is one of the most important modifiable health determinants, and preventing smoking initiation has been found to be associated with significantly reduced health risks in adulthood [7,8]. Consequently, anti-smoking efforts have been highly prioritized within public health policy in this country [9].

When developing an accurate and cost-effective anti-smoking campaign, it is particularly important to determine the factors that may be related to smoking initiation [10,11,12]. While some factors may be associated with a lower incidence of smoking, and therefore should be recognized as protective in nature, the presence of other factors may be associated with higher incidence of smoking behavior and, consequently, should be recognized as risk-factors for smoking in adolescence [13,14,15]. Previous studies conducted in Croatia and the surrounding region have identified some associations between certain sociodemographic-, educational-, familial-, and sport-related factors and the incidence of smoking in adolescents [4,16,17]. However, it is generally accepted that such associations should be examined prospectively, while focusing on specific socio-cultural circumstances known to be important for each studied cohort [18].

One group of factors that has been consistently reported to be strongly associated with smoking behavior is academic achievement (i.e., educational achievement). In brief, nearly all studies that have examined these associations in the global context have confirmed the presence of a higher incidence of smoking in adolescents who performed poorly in school [4,15,19,20]. In discussing such relationships, several explanations have been offered. First, the well-known negative influence of smoking on cognitive function has been frequently emphasized as serving as a clear ‘physiological’ background for the negative association between smoking and scholastic achievement in adolescence [21,22,23]. Although this explanation appears to be sound, the main criticism of this hypothesis is related to the relatively short time-span during which smoking occurs in adolescence. Consequently, it is unlikely that the negative influence of smoking on cognitive function would result in poor educational outcomes in adolescents who have smoked for only a few years [16]. Other theories highlight negative social influence as a more probable cause of smoking in adolescents who perform poorly in school [24,25]. In brief, children who fail academically frequently avoid school, consequently placing themselves in sociocultural circumstances where smoking is prevalent, eventually starting to smoke themselves [16,26]. Indeed, social influence is a strong determinant of adolescent behavior, an association that supports the logicality of this theory [27,28]. However, there is a lack of studies that have prospectively examined this association. This is almost certainly one of the most important reasons for the lack of knowledge regarding causality in the potential relationship between educational variables (educational-failure), and smoking in adolescence [5,16,18].

Another factor that is frequently hypothesized as being protective against smoking in adolescence is participating in sports and physical exercise [4,29,30]. In general, sports promote physiological and psychological well-being and have been found to have a significant and positive influence on the development of pro-social behaviors and self-discipline [31]. Therefore, it may logically be expected that children who are involved in sports would be less likely to smoke [32]. However, the results of studies that have examined the association between engagement in sports and smoking in adolescence have not been consistent [32,33,34]. More precisely, when investigations have compared athletes and non-athletes, the prevalence of smoking was found to be lower in adolescents actively involved in sports [33,35]. However, when the problem was examined more specifically, previous studies have reported the highest prevalence of smoking in former adolescent athletes, emphasizing the idea that quitting sports serves as a risk factor for adolescent smoking [3,4,25]. However, even then, it has not been determined which comes first. Namely, it is possible that adolescents (first) start smoking and then quit sports because of the negative influence of smoking compounded with decreases in their physiological capacity, which consequently resulted in poor sport achievement. On the other hand, it is also possible that quitting sports actually led to smoking because of changes in social influence, a hypothesis that is similar to that previously described for the association between educational failure and smoking [17].

Investigations that have examined the factors associated with smoking in adolescence have regularly emphasized the importance of prospective designs, suggesting that this methodology is the optimal approach for identifying protective/risk factors for smoking in adolescence [5,18,26]. Consequently, this study aimed to prospectively investigate the associations between educational- and sport-related factors and smoking in older adolescents in Croatia. Increasing the body of knowledge in this field will allow the most vulnerable groups of adolescents to be targeted and facilitate the development of accurate and targeted anti-smoking policies for the studied age-group. We hypothesized that the educational- and sport-related variables observed at the beginning of the studied period (i.e., at the age of 16 years) would directly influence the smoking initiation during the following two years (until the age of 18 years).

## 2. Materials and Methods

### 2.1. Participants

**Figure 1:**
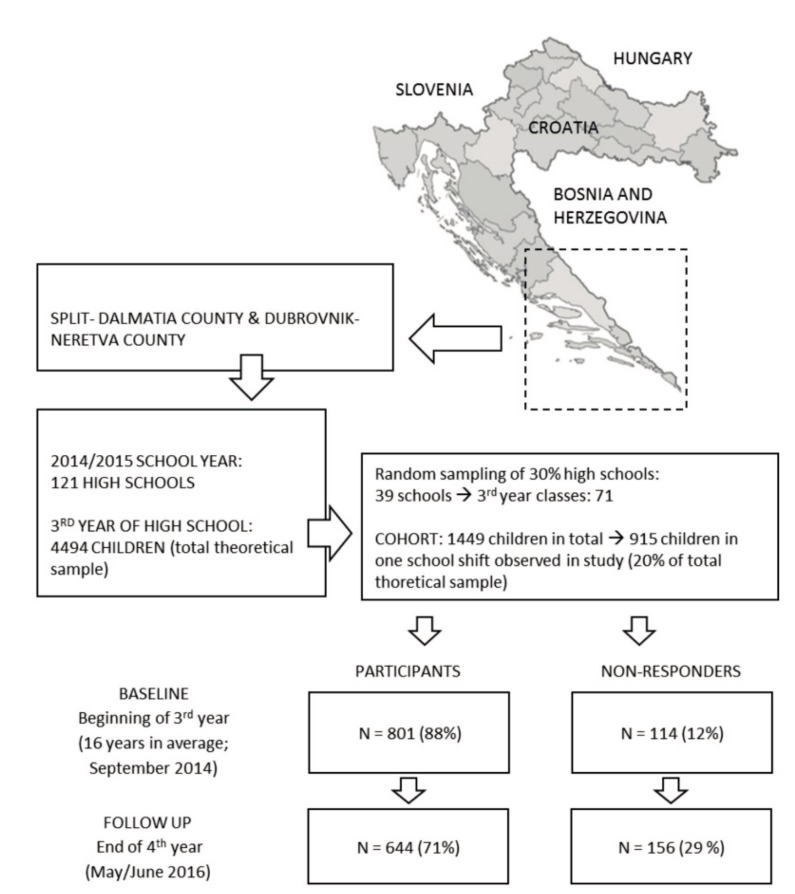
Location of the study, testing sequences, participant- and drop-out-rates.

To allow for the performance of meaningful comparisons and to fulfill gaps in the findings of previous studies, in this prospective study, we examined adolescents of the same age as those previously investigated in studies in Croatia and the surrounding region [3,4,16,17]. We included 644 adolescents from Split/Dalmatia County and Dubrovnik/Neretva County, two southern regions of Croatia (Figure 1). Because of their similar geographical location, traditions, cultural background, and heritage, we determined these two regions to be appropriate for the purposes of this investigation. Additionally, while we intended to study the potential role of sport factors in smoking prediction, it was important to study regions with similar sport traditions and in which similar types of sports are popular among adolescents (i.e., water sports, such as water polo, swimming, sailing). At baseline, the mean age of the examinees was 16 years, and they were in their third year of high school. On a basis of data on smoking prevalence previously reported in cross-sectional studies done in Split-Dalmatia county [17], with population/theoretical sample of 4494 children (see Figure 1), and level of significance of *p* < 0.05, the required sample size was 530 participants.

For the purposes of this study, we used a multistage simple random sampling method. During the first stage of sampling, all high schools in studied counties were grouped into three groups according to their attending number of pupils. Next, we randomly selected one-third of schools from each group, resulting in the selection of 39 schools overall. Finally, we decided to study the morning school shift (days within schools in the studied regions are organized in two shifts). Altogether, this sampling protocol resulted in the inclusion of 37 classes (third year of high school) and a cohort of 916 children. Study participants were anonymous, and no personal data that would allow for the identification of single participants based on their responses, were gathered. In order to follow participant responses over testing waves, the participants were asked to use a self-selected confidential code. The examiner suggested participants to use last three digits of their e-mail password as personal code, which assured anonymity and consistency (i.e., passwords are known exclusively to the owner of the e-mail account), while it is not likely that it will be changed over course of the study. The ethical protocol was approved by the ethics board of the University of Split, Faculty of Kinesiology (Ethical Approval No: 2181-205-02-05-14-005). The study design and aims were fully explained to the guardians of the children at a regular school meeting occurring at the beginning of school, and the guardians provided written consent for their child’s participation in the study. While school meetings organized at the beginning of school year are mandatory for all parents, more than 95% of all parents participated on them. For those parents who were not present, written explanation of the study with agreement was provided and sent throughout next week. Participants were tested over the following two waves: the first wave (baseline) was carried out at the beginning of the third year of high school (in late September–early October 2014; the mean age of the participants was 16 years); and the second wave (follow-up) was carried out at the end of high school (20 months later; late May–early June 2016). Participants were tested while they were in school classes occurring at the beginning of the school day (i.e., their first lesson) and while in a group of at least 10 participants. Each participant was supplied with the questionnaire and one envelope. After testing, the respondents placed the questionnaire in the envelope, sealed the envelope and placed it in closed box that was opened the next day by an investigator who was not present at testing. Testing was completed only once during each class, meaning that only the children who were at school at the moment of testing were included in the investigation.

The protocol of the study, drop-out rate, and number of tested participants are presented in Figure 1.

Of the total cohort of 915 participants, 801 were successfully tested during the first wave (88% response rate), and of those tested during the first wave, 80% were tested during the second wave. Ultimately, the drop-out rate was 29%. We performed an analysis for attrition bias for smoking status at baseline, and found no significant differences between baseline smokers and non-smokers (Chi square: 2.14, *p* > 0.10). The intracluster-correlation-coefficient calculated for baseline smoking prevalence with schools observed as clusters was 0.05, indicating appropriate within-cluster (i.e., within-school) variance [36,37].

### 2.2. Variables

The data collected in this study included sport factors, educational factors (predictors), and smoking behaviors (outcome). Additionally, since previous studies have identified strong associations between gender, age, socioeconomic status, and parental conflict and smoking behavior in adolescents, these covariates were also investigated [9,25,38,39,40]. A structured questionnaire that had been previously used and validated in cross sectional investigations completed in similar populations was used [25,41].

Sport factors: Questions on sport factors included questions on: (i) involvement in competitive sports (answered on scale consisting of three possible answers: never involved–quit–currently involved); (ii) highest sport achievement, defined as competitive achievement during sport participation (never been involved/competed–local competitions–national/international competitions); (iii) duration of sport involvement (never–less than a year–two to five years–more than five years); and (iv) participation in some kind of non-formal/non-competitive physical exercise, such as training in gym/self-exercising (no–from time to time–regularly).

Educational variables: These variables included three questions assessing: (i) grade point average (GPA) during the last semester (participants responded on a five-point scale from excellent to negative); (ii) behavioral grade, indicator of overall student’s behavior irrespective of his/her knowledge/skill achievements (excellent–average–not appropriate); and (iii) school absences (less than 10 school hours–10 to 20 h–21 to 40 h–more than 40 h).

Cigarette smoking was assessed on a six-point scale (no–quit–from time to time, but not daily–less than 10 cigarettes per day–more than 10 cigarettes per day). For the purposes of the logistic regression analysis (see later on details) participants were classified as ‘non-smokers’ (those who responded “no” or “quit”) and ‘smokers’ (the remaining answers). Additionally, smoking initiation was identified for all participants who were evidenced as ‘non-smokers’ at baseline and as ‘smokers’ at follow-up.

Covariates (i.e., confounding factors) included participant age, gender, socioeconomic status (SES: under average–average–above average), and conflict with parents (four-point scale from “no conflict at all” to “frequently in conflict with parents”).

### 2.3. Statistics

Descriptive statistics included counts and percentages. Differences between smokers and non-smokers were identified in predictor variables and covariates by Mann-Whitney test (MW) or Chi square test (χ^2^). To evaluate the relationships between predictors and outcomes, a series of logistic regression models were generated to produce odds ratios (ORs) and their corresponding 95% confidence intervals (CIs). The following three outcomes were evaluated: baseline smoking status, follow-up smoking status, and smoking initiation. In addition to the crude logistic regression analysis (unadjusted regression models), the following adjusted models were also calculated: (i) model adjusted for age and gender; (ii) model adjusted for age, gender and SES; and (iii) model adjusted for age, gender, SES, and parental conflict. The logistic regression generated for smoking initiation included participants who were non-smokers at baseline. The *p*-level of 95% was applied. Statistica ver. 12.0 (Statsoft, Tulsa, OK, USA) was used for all calculations.

## 3. Results

The prevalence of smoking increased between the testing waves (265, 41%; and 312, 49% for baseline and follow-up testing, respectively). The increase in smoking prevalence was evidently greater in females (110, 37%; and 148, 49%, respectively) than in males (154, 44%; and 164, 48% smokers for baseline and follow-up, respectively).

Significant differences between smokers and non-smokers were identified in sport-participation at both testing waves (χ^2^ = 21.86 and 14.01, both *p* < 0.01, for baseline and follow-up, respectively), with the highest prevalence of smoking for those who quit sport. Non-smokers achieved better GPAs (MW = 7.62 and 5.18, both *p* < 0.01), better behavioral grades (MW = 4.28 and 3.26, both *p* < 0.01), and had less school absences (MW = 3.9 and 3.49, both *p* < 0.01 for baseline and follow-up respectively) than smokers (Supplementary Table S1).

Increased odds of being smokers at baseline were observed in adolescents with lower GPAs (OR: 2.10, 95% CI: 1.61–2.55), lower behavioral grades (1.80, 1.10–2.89), and more school absences (1.40, 1.19–1.69). Quitting sports was identified as a risk-factor for smoking at baseline (2.07, 1.31–3.32). Almost identical associations as those observed for baseline were evidenced between the evaluated educational variables and smoking at follow-up (GPA: 1.59, 1.31–1.94; school absence: 1.30, 1.08–1.58; behavioral grade: 1.57, 1.03–2.41), and quitting sports and smoking at follow up (1.66, 1.09–2.56) (Table 1).

Higher competitive achievement is found to be protective factor against smoking initiation during the study period (0.45, 0.21–0.91) (Table 2).

## 4. Discussion

The aim of this study was to evaluate possible associations between educational and sport factors and smoking prevalence and initiation in older adolescents from Croatia. The results revealed that educational variables (overall achievement in school) were strongly associated with smoking prevalence at study baseline and follow-up. However, the assessed educational variables were not found to be associated with smoking initiation over the course of the study. Participation in sports was identified to be associated with smoking, with greater odds of smoking identified in adolescents who practiced in and then quit sports. Finally, higher competitive achievement in sport was determined to be a protective factor against smoking initiation in older adolescents.

### 4.1. Educational Factors and Smoking

Educational achievement was strongly associated with smoking prevalence. In brief, all evaluated educational variables were negatively associated with smoking at both baseline and follow-up. Therefore, our results are in accordance with the results of previous reports in which poor scholastic achievement was often identified in children who smoked [4,15,19,20]. Meanwhile, we did not identify the presence of an association between the studied educational factors and smoking initiation over the course of the study. Therefore, although we, in general, filled the existing gap with regards to experimental design (i.e., previous studies have been mostly cross-sectional, while we observed the association prospectively), this investigation did not contribute to the body of knowledge regarding the possible cause-effect relationship between educational variables and smoking initiation.

There are several possible explanations of the identification of the absence of an association between educational variables and smoking initiation, despite strong cross-sectional associations between scholastic variables and smoking prevalence. First, we must note that the sample of participants included in this investigation were adolescents from different high schools. Therefore, it is possible that differences in the “quality of schools” and, consequently, differences in the relative evaluation of scholastic achievement within schools may have resulted in a certain bias, thereby prohibiting us from evaluating the influence of scholastic variables on smoking initiation in the studied group of adolescents. Second, it is possible that each of the two theories (i.e., ‘physiologically based’ and ‘socially based’; see Introduction for details) proposed in an attempt to explain causality within the association between educational-achievement and smoking-initiation actually hold true for some of adolescents who started to smoke between 16 and 18 years of age [21,22,23,24,25]. Precisely, for some adolescents who initiated smoking during this period, educational failure could be ‘the cause’, while for the others it could be ‘the effect’ of smoking initiation. Next, it is also possible that both smoking and educational failure are generated by same mechanism, as explained by the theory of problem behavior. In brief, some adolescents have general psychosocial tendency for unconventionality, and therefore ‘problematic behaviors’ in some participants could appear in tandem, simultaneously resulting in (i) poor scholastic achievement and (ii) smoking [42]. Finally, and what we believe that should be judged as most probable explanation, it is possible that the association between educational variables should be evaluated at an earlier age, when most adolescents actually started to smoke. Of course, one can argue that scholastic achievement was not entirely objectively evaluated because the data were self-reported. Although this should be considered an important limitation of the study, we believe that employing strict participant anonymity decreased the possibility that the participants responded dishonestly.

### 4.2. Sport Factors and Smoking

Previous cross-sectional studies have reported the presence of different associations between sport-related factors and smoking in older adolescents. In brief, the prevalence of smoking has been found to be lower among adolescents actively involved in sports, but those who were once involved in sports and then quit were observed to be more likely to be smokers than those who were never involved in sports [3,26]. Therefore, the results regarding the high prevalence of smoking in participants who quit sports supported the results of previous studies in which quitting sports was highlighted as a risk factor for smoking in adolescence [4,16,25]. However, participation in sports was not associated with smoking initiation during the studied period. Therefore, it is almost certain that in the studied adolescents, a true cause-effect relationship between quitting sports and smoking initiation may be observed at an earlier age.

Competitive achievement in sports was identified as a factor that influenced smoking initiation during the study period, with a lower likelihood of smoking initiation observed in adolescents who achieved better competitive results. Although this was the only significant correlation between observed factors and smoking initiation and therefore these results (i.e., protective effect of competitive achievement against smoking initiation) could be considered as being accidental to some extent, similar associations between of sport-competitive achievement on smoking and smoking-initiation are noted in some previous reports [3,16,18]. In brief, Tahiraj et al. identified lower prevalence of smoking in boys who achieved better competitive results in sport, while Zenic et al. observed there to be a higher risk of smoking in girls who practiced sports but had lower sport achievement [3,16]. Similarly, in a very recent study those children who were involved in sports competitions but with lower competitive results have been found as being at higher risk for smoking initiation than their peers who were never involved in sports [18]. In general, two possible hypotheses regarding the direction of causality were proposed. First, it was hypothesized that a lack of sport success could cause a certain frustration, resulting in smoking. However, it was also possible that smoking negatively influenced the performance of young athletes [4,16,25]. Evidently our results are supportive to these findings and may help to eliminate the doubt of cause-effect relationship between sport-success and smoking in older adolescents. Briefly, it was evident that a lack of sport achievement may be considered the cause of smoking initiation in older adolescents and not vice-versa. Additionally, children who were successful in sports by the age of 16 years were less likely to smoke during the following two years (until the age of 18).

What is remains unknown is the mechanism by which the “positive influence” of sport success affects smoking in adolescence. Namely, there is a certain possibility that children who achieved more success in sports are well aware of the negative influence of smoking on their physical capacities and, therefore, avoided cigarettes [43]. However, there is also a possibility that children who performed well in sports demonstrated strong self-discipline, which resulted in both (i) sport success and (ii) avoidance of smoking. This association should be investigated in the future using a qualitative approach. In doing so, some additional, potentially confounding variables should be observed. One such factors is definitively ‘peer-influence’ which was not taken into account in this investigation. In brief, adolescents who are exposed to positive social influences (i.e., non-smoking peers who participate in sports and achieve good results) are naturally under the influence of positive role-models, which consequently could influence both (i) avoidance of smoking; and (ii) higher involvement and competitive achievement in sports.

### 4.3. Limitations and Strengths

The first limitation of this investigation comes from the use of self-report of data. Consequently, participants may have been more likely to provide socially desirable answers. Indeed, data were self-reported, but as strict anonymity was ensured, and the study was conducted in a country where smoking is socially acceptable, the possibility that participants did not respond honestly is lower. The most important limitation arises from the fact that the majority of participants initiated smoking before the age of 16 years, and, therefore, the generalizability of the results regarding the prediction of smoking initiation is limited solely to adolescents who initiated smoking during late adolescence (16 to 18 years of age).

This study is one of the first to prospectively investigate the predictors of smoking in southeastern Europe and in a country with an alarmingly high prevalence of smoking in adolescence. Additionally, a significant strength of the investigation was a relatively high retention rate, which allowed us to objectively discuss the observed results. Therefore, although we are aware that our results cannot fully elucidate this association, we believe that this study contributes to existing knowledge in this field.

## 5. Conclusions

Although most of the adolescents evaluated in this study started to smoke cigarettes before the age of 16 years, the prevalence of smoking has continued to rise, even during the two years under study. This is particularly evident in females and, in future studies, the reasons for dramatic increase in prevalence of smoking among females relative to males should be evaluated. In such investigations, potential gender-specific factors such as satisfaction with physical appearance or self-perception of body-weight, or both, should be observed as potential predictors of smoking initiation.

Since educational achievement was not found to be associated with smoking initiation in the studied adolescents, the causal relationship between scholastic variables and smoking remains unclear. The results of this study suggest that participation in sports is not, per se, a factor that contributes to smoking initiation in older adolescents. Meanwhile, the association between sports and smoking should be evaluated while observing different facets of sport involvement that explain various aspects of practicing sports in adolescence (i.e., time of involvement, achieved result, type of sport, motives for participation in sport, etc.). Future studies should explore the relationships between variables and smoking initiation at a younger age, when most of studied adolescents initiated smoking.

## Figures and Tables

**Table 1:** Correlates of smoking at baseline and follow-up for 265 (41%) and 312 (49%) being smokers at baseline and follow-up, respectively.

	Baseline (Model 3)		Follow-Up (Model 3)	
	OR	95% CI	OR	95% CI
**Grade Point Average (cont)**	2.01 *	1.61–2.55	1.59 *	1.31–1.94
**School absence (cont)**	1.40 *	1.19–1.69	1.30 *	1.08–1.58
**Behavioral grade (cont)**	1.80 *	1.10–2.89	1.57 *	1.03–2.41
**Participation in sport**				
Currently involved	Ref		Ref	
Quit	2.07 *	1.31–3.32	1.66 *	1.09–2.56
No, never	0.98	0.61–1.59	0.87	0.56–1.39
**Recreation (physical exercising other than sport)**				
Regularly	Ref		Ref	
From time to time	1.30	0.89–2.00	1.29	0.86–1.92
Not involved	1.40	0.90–2.03	1.48	0.93–2.35
**Experience in sport**				
Never involved	Ref		Ref	
Less than a year	0.52 *	0.29–0.89	1.04	0.52–2.09
2–5 years	1.31	0.73–2.37	1.83 *	1.09–3.08
More than 5 years	1.33	0.92–1.92	1.32	0.79–2.20
**Achieved result in sport**				
Never involved/Never competed	Ref		Ref	
Regional level	1.23	0.86–1.79	1.05	0.74–1.51
National/international level ^¥^	1.53	0.50–4.68	0.22	0.05–1.02

* denotes statistical significance of *p* < 0.05; Ref: reference value; cont: continuous variable; Model 3: logistic regression adjusted for gender, age, socioeconomic status and conflict with parents; OR: odds ratio; CI: confidence interval; ^¥^ participants who reported National and International results are grouped together.

**Table 2:** Correlates of smoking initiation for 47 participants who initiated smoking during the course of the study (12% of 379 adolescents who did not smoke at study baseline).

	Crude		Model 1		Model 2		Model 3	
	OR	95% CI	OR	95% CI	OR	95% CI	OR	95% CI
**Grade Point Average (cont)**	0.87	0.62–1.22	1.03	0.72–1.48	1.03	0.72–1.48	1.00	0.69–1.45
**School absence (cont)**	1.22	0.91–1.66	1.21	0.89–1.65	1.21	0.89–1.65	1.19	0.87–1.63
**Behavioral grade (cont)**	0.57	0.21–1.55	0.95	0.34–2.69	0.95	0.34–2.69	0.85	0.29–2.45
**Participation in sport**								
Currently involved	Ref		Ref		Ref		Ref	
Quit	1.05	0.53–2.10	0.79	0.38–1.63	0.79	0.38–1.63	0.76	0.37–1.59
No, never	0.95	0.48–1.89	0.70	0.34–1.44	0.70	0.34–1.44	0.69	0.34–1.44
**Recreation (physical exercising other than sport)**								
Regularly	Ref		Ref		Ref		Ref	
From time to time	2.41 *	1.15–5.07	1.91	0.89–4.12	1.94	0.89–4.19	1.92	0.89–4.15
Not involved	2.10	0.92–4.80	1.57	0.67–3.67	1.58	0.67–3.69	1.58	0.67–3.71
**Experience in sport**								
Never involved	Ref		Ref		Ref		Ref	
Less than a year	0.26	0.05–1.25	0.25	0.05–1.19	0.25	0.05–1.19	0.22	0.05–1.09
2–5 years	1.23	0.59–2.57	1.30	0.61–2.78	1.31	0.61–2.81	1.27	0.59–2.72
More than 5 years	0.71	0.34–1.45	0.88	0.42–1.87	0.89	0.42–1.88	0.89	0.42–1.89
**Achieved result in sport**								
Never involved/Never competed	Ref		Ref		Ref		Ref	
Regional level	0.62	0.35–1.08	0.63	0.35–1.12	0.63	0.35–1.12	0.64	0.36–1.16
National/international level ^¥^	0.42 *	0.18–0.91	0.45 *	0.21–0.90	0.45 *	0.21–0.90	0.45 *	0.21–0.91

* denotes statistical significance of *p* < 0.05; Crude: unadjusted logistic regression; Model 1: logistic regression adjusted for gender and age; Model 2: Model 1 + socioeconomic status; ^¥^ participants who reported National and International results are grouped together.

## Supplementary Materials

The following are available online at www.mdpi.com/1660-4601/14/4/446/s1. Table S1: Baseline and follow-up characteristics with differences between smokers (S) and non-smokers (NS) (Chi square test—χ^2^; Mann Whitney test—MW).
