# Supplementary material for: Prospective Analysis of the Influence of Sport and Educational Factors on the Prevalence and Initiation of Smoking in Older Adolescents from Croatia
Source: Int J Environ Res Public Health. 2017 Apr 20;14(4):446. doi: 10.3390/ijerph14040446 (PMC5409646; doi:10.3390/ijerph14040446)
Supplement: Supplementary file 1 [file ijerph-14-00446-s001.pdf]

Supplementary Data: Prospective Analysis of the Influence of Sport and Educational Factors on the Prevalence and Initiation of Smoking in Older Adolescents from Croatia

Natasa Zenic [1], Djivo Ban [1,2], Sanja Jurisic [3], Mladen Cubela [3], Jelena Rodek [1], Ljerka Ostojic [1,3,4], Mario Jelicic [1], Antonino Bianco [5], and Damir Sekulic [1,6,*]

Supplementary Table 1

Baseline and follow-up characteristics with differences between smokers (S) and non-smokers (NS) (Chi square test – χ^2; Mann Whitney test – MW)

	Baseline			Follow up		
	NS; n = 379 (59%)	S; n = 265 (41%)	χ^2 (p)	NS; n = 332 (51%)	S; n = 312 (49%)	χ^2 (p)
	F (%)	F (%)		F (%)	F (%)	
Participation in sport			21.86 (0.01)			14.01 (0.01)
Currently involved	84 (22.1)	43 (16.31)		72 (21.69)	54 (17.31)	
Quit	142 (37.57)	148 (56.03)		128 (38.55)	166 (53.21)	
No, never	153 (40.33)	73 (27.66)		132 (39.76)	92 (29.49)	
Missing	0 (0)	0 (0)		0 (0)	0 (0)	
Recreation (physical exercising other than sport)			1.07 (0.58)			3.17 (0.21)
Regularly	92 (24.31)	55 (20.57)		84 (25.3)	62 (19.87)	
From time to time	188 (49.72)	137 (51.77)		166 (50)	160 (51.28)	
Not involved	98 (25.97)	73 (27.66)		82 (24.7)	90 (28.85)	
Missing	1 (0)	0 (0)		0 (0)	0 (0)	
Gender			3.76 (0.04)			0.32 (0.56)
Female	188 (49.72)	111 (41.84)		150 (45.18)	148 (47.43)	
Male	191 (50.28)	154 (58.16)		182 (54.82)	164 (52.56)	
Missing	0 (0)	0 (0)		0 (0)	0 (0)	
			MW (p)			MW (p)
Experience in sport			-0.83 (0.41)			-0.07 (0.94)
Never involved	65 (17.13)	21 (7.8)		50 (15.06)	34 (10.9)	
Less than a year	29 (7.73)	26 (9.93)		32 (9.64)	24 (7.69)	
2-5 years	111 (29.28)	101 (38.3)		94 (28.31)	120 (38.46)	
More than 5 years	174 (45.86)	117 (43.97)		156 (46.99)	134 (42.95)	
Missing	0 (0)	0 (0)		0 (0)	0 (0)	

Achieved result in sport			-1.65 (0.10)			0.98 (0.33)
Never involved/Never competed	142 (37.57)	83 (31.21)		114 (34.34)	110 (35.26)	
Regional level	163 (43.09)	120 (45.39)		142 (42.77)	142 (45.51)	
National level	65 (17.13)	55 (20.57)		64 (19.28)	56 (17.95)	
International level	8 (2.21)	6 (2.13)		12 (3.61)	2 (0.64)	
Missing	1 (0)	1 (0)		0 (0)	2 (0.01)	
Socio-economic status			1.07 (0.28)			0.1 (0.92)
Below average	4 (1.1)	13 (4.96)		12 (3.61)	6 (1.92)	
Average	329 (86.74)	219 (82.98)		276 (83.13)	272 (87.18)	
Above average	44 (11.6)	32 (12.06)		42 (12.65)	34 (10.9)	
Missing	2 (0.01)	0 (0)		2 (0.01)	0 (0)	
Conflict with parents			-3.9 (0.01)			-3.76 (0.01)
Never	65 (17.13)	38 (14.18)		64 (19.28)	38 (12.18)	
Rarely	178 (46.96)	90 (34.04)		146 (43.98)	120 (38.46)	
From time to time	119 (31.49)	106 (40.43)		106 (31.93)	122 (39.1)	
Frequently	17 (4.42)	30 (11.35)		16 (4.82)	32 (10.26)	
Missing	0 (0)	0 (0)		0 (0)	0 (0)	
Grade Point Average			-7.62 (0.01)			-5.18 (0.01)
Excellent	82 (21.55)	21 (7.8)		68 (20.48)	32 (10.26)	
Very good	203 (53.59)	111 (41.84)		172 (51.81)	140 (44.87)	
Average	84 (22.1)	103 (39.01)		76 (22.89)	114 (36.54)	
Above average/Insufficient	2 (0.55)	15 (5.67)		14 (4.22)	26 (8.34)	
Missing	2 (0.01)	1 (0)		0 (0)	0 (0)	
School absence			-3.9 (0.01)			-3.49 (0.01)
Less than 10 school hours	128 (33.7)	60 (22.7)		110 (33.13)	76 (24.36)	
10-20 hours	163 (43.09)	107 (40.43)		146 (43.98)	124 (39.74)	
21-40 hours	71 (18.78)	86 (32.62)		62 (18.67)	98 (31.41)	
More than 40 hours	17 (4.42)	11 (4.26)		14 (4.22)	14 (4.49)	
Missing	0 (0)	0 (0)		0 (0)	0 (0)	
Behavioral grade			-4.28 (0.01)			-3.26 (0.01)
Good	348 (91.71)	212 (80.14)		302 (90.96)	256 (82.05)	
Average	27 (7.18)	45 (17.02)		24 (7.23)	50 (16.03)	
Above average/Insufficient	4 (1.1)	8 (2.84)		6 (1.81)	6 (1.92)	
Missing	0 (0)	0 (0)		0 (0)	0 (0)	
